# Aerobic Exercise-Assisted Cardiac Regeneration by Inhibiting Tryptase Release in Mast Cells after Myocardial Infarction

**DOI:** 10.1155/2021/5521564

**Published:** 2021-06-08

**Authors:** Mohammad Bayat, Sufan Chien, Farzaneh Chehelcheraghi

**Affiliations:** ^1^Department of Biology and Anatomical Sciences, School of Medicine, Shahid Beheshti University of Medical Sciences, Tehran, Iran; ^2^Price Institute of Surgical Research, University of Louisville, and Noveratech LLC of Louisville, USA; ^3^Anatomical Sciences Department, Lorestan University of Medical Sciences, Khorramabad, IR, Iran

## Abstract

**Background:**

Cardiovascular disease (CVD) contributes critically to the mortality, morbidity, and economic problem of illness globally. Exercise is a share of everyone's life. Some evidence-based studies have frequently shown a progressive correlation between physical activity and good health.

**Objective:**

The effects of daily exercise on cardiomyocyte size, collagen content (fibrosis), and releasing mast cells (MCsʼ) tryptase of the model of myocardial infarction (MI) were assessed.

**Methods:**

40 rats were coincidentally spread into sham+inertia (control), sham+exercise, infarction+inertia, and infarction+exercise groups. An experimental model of acute MI was induced in infarction groups. One week after surgery, exercising groups were allowed to an aerobic exercise program for six weeks. At the endpoint of the study, all examinations were performed.

**Results:**

We found lesser fibrosis in sham+exercise and infarction+exercise groups compared to sham+inertia and infarction+inertia groups, respectively (*p* = 0.023, *p* = 0.001). Also, infarction groups were significantly lower than sham groups (*p* < 0.05) and the infarction+exercise group was significantly lower than the infarction+inertia group (*p* < 0.05). The effect of exercise on MCs while increased MC density and degranulation occur at the site of fibrosis, we demonstrated that exercise decreases both total MC density and degranulation in both sham and infarction groups (*p* < 0.05). Immunohistochemistry examinations were significantly higher expression of MCsʼ tryptase in infarction groups than sham groups (*p* < 0.05, *p* < 0.0001).

**Conclusion:**

Exercise improves fibrosis and cardiac function in both healthy and MI rats by inhibiting released MCsʼ tryptase.

## 1. Introduction

Cardiovascular diseases (CVDs) continue to be the chief source of mortality in the USA, in charge of more than 840,000 fatalities in 2016 [1]. More than 30% of CVD-connected fatalities are ascribed to myocardial infarction (MI) [2]. The signaling paths that are triggered subsequent cardiomyocyte death start acute amendatory alterations in the heart that could be fragmented into first and delayed steps. The first step includes the substitution of necrotic cells with fibrotic scar establishment, lengthening of cardiomyocytes, and diminishing of the infarct area [3, 4]. The delayed step will move forward indeterminately and finally happens in heart failure [3]. Though fibrosis is a firstly positive course of healing, continuing addition of collagen fibers conducts to constant tissue renovation and substantial organ damage [5]. Results of many clinical trials of heart rehabilitation including exercise after MI determined that ordinary exercise (practice) diminishes the danger of global death and CV death [6]. Mast cells (MC) are natural defensive cells existent in almost all human organs with crucial actions in allergic illness and host defense. MC amounts are commonly amplified at places of fibrosis. They are powerful, local, effector cells creating intermediaries that control the fibrosis course. The character of the releasing molecules created by MCs rests on circumstance signals, and they could be both pro- and antifibrotic. MC releasing molecules could excite fibroblast multiplying, suggesting that fibroblasts are the main actors in fibrosis. One MC creation, the serine protease tryptase, is recognized to trigger protease-activated receptor 2 (PAR2) and conduct to multiplying of fibroblasts and induce fibroproliferative activities in human fibroblasts [7]. MCs have been frequently connected to the pathogenesis of fibrosis in the heart, but these results are debatable [8]. We for the first time will identify potential roles of MCs and the MCsʼ tryptase in MI under influence of exercise and will provide opportunities to manipulate MCs to inhibit or reduce damaging fibrosis in the heart. Vital beneficial chances can rise from a better comprehension of the impact of MCs, with the capability to majorly change permanent fibrotic courses in CVDs.

## 2. Materials and Methods

### 2.1. Animals and Study Design

All procedures were permitted by the University Board on medical ethics of Lorestan University of Medical Sciences (LUMS.REC.1396.257). First, 40 adult male Wistar rats were held in a standard animal home with free access to food and water. Next, they were randomized into sham+inertia (control), sham+exercise, infarction+inertia, and infarction+exercise groups. After that, an experimental model of MI was induced in infarction groups. One week after surgery, exercising groups were allowed to exercise for six weeks. At the study endpoint (7 weeks after induction of MI), all examinations were performed.

### 2.2. Model of Acute Myocardial Infarction

Infarction animals were submitted to a constant tying of the anterior interventricular artery (AIA). Under profound anesthesia (ketamine (50 mg/kg body wt) and xylazine (5 mg/kg body wt)), the heart was exposed through left thoracotomy in the left 5 intercostal space. In the infarction group, a 9–0 nylon suture (SUPA, Iran) was positioned around the beginning of AIA, and the artery was tied. Sham groups abided by the same surgery, excepting that the suture around AIA was not applied. Next, the heart was replaced with its anatomical location and the chest instantly sealed [1].

### 2.3. Exercise Protocol

One week after surgery, rats from exercise groups participated in a training protocol. The exercise protocol included five sessions of interval running per week on a treadmill (animal research veterinary treadmill 47300 Ugo Basile, Italy) for 6 following weeks. The running speed was primarily 10 m/min and was gradually increased to 22 m/min to do incremental treadmill exercise tests for 5 days (equal to 50-90% VO2max). Following the familiarization period, animals underwent an incremental treadmill exercise test, whose primary speed was set at 10 m/min followed by a 22 m/min increase every 3 min, until exhaustion (active recovery at 22 m/min, equal to 60-75% VO2max). The animals performed the exercise test without any encouragement [2].

### 2.4. Echocardiography

Echocardiography assessed cardiac function noninvasively (software: ML750 Power Lab/4sp AD Instruments GE-Vingmed Ultrasound, USA); the animals underwent mild anesthesia with 5% isoflurane and were evaluated by an echo device equipped with a 14-18 MHz heart probe. Echocardiographic parameters were obtained based on the main axis of the heart. Heart failure with an ejection fraction < 55% is clear. Calculation of cardiac output was estimated as (end − diastolic volume–end − systolic volume) × heart rate (ml/min) [3] (Table 1).

### 2.5. Examinations of Cardiomyocyte Diameter, Cardiomyocyte Hypertrophic Index, Cardiac Fibrosis, and Total MC Density and Degranulation (Activation)

At 1 week after MI, the animals were sacrificed to collect heart tissue. The body weight and heart of rats were measured. The hearts were fixed in 10% formalin, then sampled from the border region of myocardium infarction of the left ventricle of each heart. Then, 5 *μ*m thick sections were stained with the hematoxylin and eosin method (H&E) and were examined with stereological (physical dissector method) and immunohistochemical methods. The cardiomyocyte diameter (*μ*m) was calculated using Image J software (NIH, USA) in H&E stained slides. Inclusion measures for approving cardiomyocytes were as follows: (1) the cardiomyocytes which had a nucleus, a clear cell boundary, and around or rectangular shape (length/width ratio < 1.5%). The cardiomyocyte diameter of 6 cells per each field of 6 fields (totally 30 cells per each ratʼs heart) was counted. The histological sections were stained with Van Gieson's method for examining fibrosis, and the red-colored zones in the muscular tissue represented collagen fibers. The degree of cardiac fibrosis was calculated through the below formula, using an Image J software, based on a color histogram [4, 5].

(1) $$\begin{array}{c} \text{Percentage of fibrosis area}=\text{the fibrosis }\left(\text{connective tissue}\right)\text{ surface area }\left(\mu {\text{m}}^{2}\right)/\text{total area of the cardiomyocytes }\left(\mu \text{m}\right)\times 100. \end{array}$$

For the MCs, the sections were stained with 1% toluidine blue. We calculated a total number of (1) intact (total MCs density, Nv) MCs and (2) degranulated MCs (Nv of degranulated MCs) according to the physical dissector method:

(2) $$\begin{array}{c} \text{Nv}=\frac{\sum Q}{a/f}\times h\times \sum P, \end{array}$$

where “*ΣQ*” is the count of MCs, “*a*/*f*” is the frame area, “*h*” is the height of the dissector, and “*ΣP*” is the number of frames counted in all of the fields. Next, we calculated the total count of MCs (*N*) in each rat:

(3) $$\begin{array}{c} N=\text{numerical density }\left(NV\right)\times \text{total volume }\left(V\right). \end{array}$$

Degranulated MCs were diagnosed by released granules. The presence of degranulation allowed us to discriminate between intact and activated MCs, which represent two types of MC. Inactivated MCs have an intact cell membrane without any granules. Inactivated MCs (degranulated MC), they released some granules.

### 2.6. Immunohistochemistry Analyses

Immunohistochemistry analysis was performed as previously described. Briefly, anti-MC tryptase antibody (AA1) (ab2378, Abcam, USA) and rabbit anti-mouse IgG-TRITC (Santa Cruz, USA) were used to detect positive tryptase MCs in the hearts. The sections were observed using fluorescence microscopy [4]. The Image J software was used for quantitative analysis of cell density of the tryptase-positive MCs. Four sections from four rats in each group were examined. Besides, probable densities of the fluorescent protein including low expression and high expression of positive tryptase MCs in each section were calculated as a percentage of the immunopositivity through the following formula [5]:

(4) $$\begin{array}{c} \text{Percentage of immunopositivity}=\frac{\text{selected pixels}\times 100}{\text{total pixels}}. \end{array}$$

### 2.7. Statistical Analysis

The data are presented as the mean ± standard error of the mean (SEM), and they were examined by using a 3-way analysis of variance (ANOVA), followed by the Tukey test. A *p* value < 0.05 was statistically significant. Relationships among variables were analyzed by linear regression and Pearson correlation coefficient methods.

## 3. Results

### 3.1. Echocardiographic and Anatomical Data

All rats tolerated surgery, and there was no infection or exudate at the site of surgery. Table 1 shows the mean ± SEM of anatomical data in the studied groups. There were no significant differences in systolic and diastolic blood pressures among the studied groups. We observed significant differences in heart weight, body weight, and the ratio of heart weight to body weight, and cardiomyocyte diameter among studied groups. Cardiomyocyte diameter of infarction groups was significantly higher than sham groups (*p* < 0.05). At the same time, the cardiomyocyte diameter of the infarction+exercise group was significantly lower than the infarction+inertia group (*p* < 0.05).

### 3.2. Effect of Exercise on Cardiac Fibrosis

**Figure 1:**
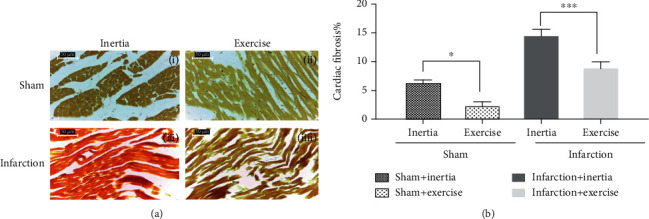
(a) Shows histological slides of cardiac fibrosis in (i) sham+inertia, (ii) sham+exercise, (iii) infarction+inertia, and (iiii) infarction+exercise groups which stained with Van Gieson's staining method. In (b), percentage of cardiac fibrosis in studied groups is expressed as the mean ± SEM and compared with 3-way ANOVA and Tukey tests; ^∗^*p* < 0.05 and ^∗∗∗^*p* < 0.001.

Figure 1(a) shows histological slides of cardiac fibrosis in (i) sham+inertia, (ii) sham+exercise, (iii) infarction+inertia, and (iiii) infarction+exercise groups which stained with Van Gieson's staining method.  Figure 1(b) represents significant decreases in cardiac fibrosis of exercise groups, compared to inertia groups. In detail, the Tukey test showed significant decreases in cardiac fibrosis of sham+exercise and infarction+exercise groups, compared to sham+inertia and infarction+inertia groups (*p* = 0.0001, *p* = 0.004).

### 3.3. Effect of Exercise on the Total MC Density and Degranulated MCs

**Figure 2:**
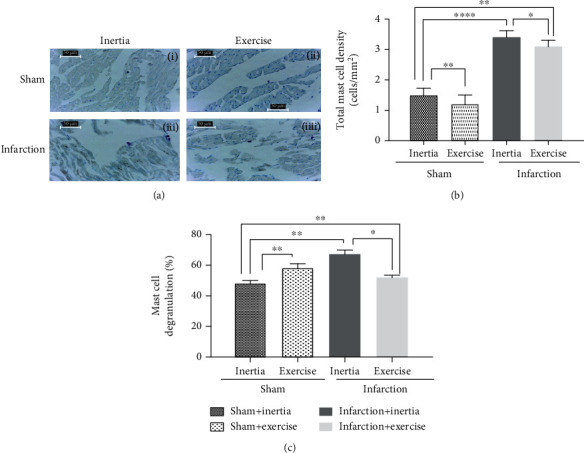
(a) Shows histological slides of mast cells in (i) sham+inertia, (ii) sham+exercise, (iii) infarction+inertia, and (iv) infarction+exercise groups stained with toluidine blue staining method. (b) and (c) show mean ± SEM of total mast cell density, and degranulation of studied groups compared with 3-way ANOVA, and Tukey tests. ^∗^*p* < 0.05, ^∗∗^*p* < 0.01, and ^∗∗∗∗^*p* < 0.0001.

Figure 2(a) shows histological slides of MCs in sham+inertia, sham+exercise, infarction+inertia, infarction+exercise, and infarction groups.  Figure 2(b) shows mean ± SEM of total MC density and degranulated MCs of studied groups compared with three-way ANOVA and Tukey tests. While myocardial infarction significantly increased total and activity MC density, exercise significantly decreased the activity of mast cells in all groups (except infarction+inertia) that were trained in exercise. In detail, the infarction+inertia group significantly increased total MC density compared to sham+inertia and infarction+exercise groups (Tukey test, *p* = 0.009, *p* = 0.024). The infarction+exercise group significantly increased MC cell density compared to sham+inertia (Tukey test, *p* = 0.009). Sham+inertia significantly increased total number MC density compared to the sham+exercise group (Tukey test, *p* = 0.0063). Activated (degranulated) MCs were significantly higher in infarction+intertia than sham+inertia, and infarction+exercise groups (Tukey test, *p* = 0.004, *p* = 0.011). Sham+exercise and infarction+exercise groups significantly increased degranulated MCs in comparison with sham+inertia groups (Tukey test, both *p* = 0.001).

### 3.4. Relationships between Cardiomyocyte Size and MCs

**Figure 3:**
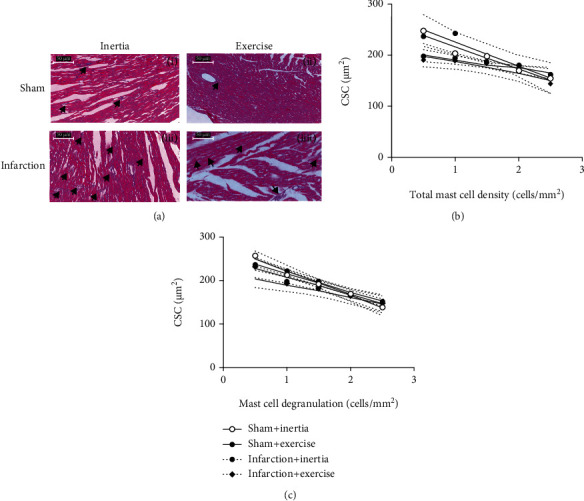
(a) Shows histological slides of cardiomyocytes of (i) sham+inertia, (ii) sham+exercise, (iii) infarction+inertia, and (iv) infarction+exercise groups stained with hematoxylin and eosin method. Black arrow demonstrated cardiomyocyte disarray. (b) Shows correlations between cardiomyocyte diameter and total MC density. (c) Shows a correlation between cardiomyocyte diameter and MC degranulation. The solid line is the line of finest fit on linear regression, and the dotted lines represent the 95% confidence. Linear regression analysis indicated an opposed correlation between the cardiomyocyte diameter and total MC density and degranulation from exercise training-induced cardiac hypertrophy.

Figure 3(a) shows histological slides of cardiomyocytes in sham+inertia, sham+exercise, infarction+inertia, and infarction+exercise groups stained with the H&E method. (b) shows correlation between cardiomyocyte diameter and total MC density. (c) shows correlation between cardiomyocyte diameter and MC degranulation. Both graphs in (b) and (c) suggest that due to exercise, cardiomyocyte diameter is inversely associated with total MC density and degranulation, and there were strong correlations for both cases (*r*^2^ = 0.555.8, *p* < 0.0001; *r*^2^ = 0.6150, *p* < 0.0001, respectively). Moreover, the relationships of cardiomyocyte diameter due to MC degranulation (Figure 3(c)) were direct but less correlated (*r*^2^ = 0.1634, *p* = 0.0423 in total density; *r*^2^ = 0.1367, *p* = 0.0312 in degranulation). These results revealed the opposite role of MCs in physiological-induced (sham) and pathological-induced (infarction) cardiac hypertrophy.

### 3.5. Relationships between Cardiac Fibrosis and MCs

**Figure 4:**
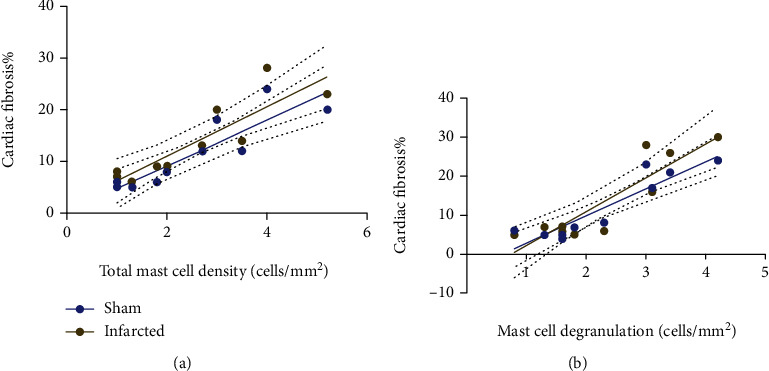
(a) Shows correlations between cardiac fibrosis and total mast cell density. (b) Shows correlations between cardiac fibrosis value and mast cell degranulation. The solid line represents the best fit of linear regression in sham groups, while the dotted line shows the best fit of linear regression in infarction groups. Linear regression is presented by solid line and indicated the direct relationship of the cardiac fibrosis value and total MC density and degranulated MC in the sham groups (*r*^2^ = 0.1706 and *r*^2^ = 0.1847, respectively). But there was no correlation in the infarction groups (*r*^2^ = 0.10112 and *r*^2^ = 0.03386).

Figure 4(a) shows correlations between the percentage of cardiac fibrosis and total MC density.  Figure 4(b) shows correlations between the percentage of cardiac fibrosis and MC degranulation.

### 3.6. Findings of Immunohistochemistry Examination

**Figure 5:**
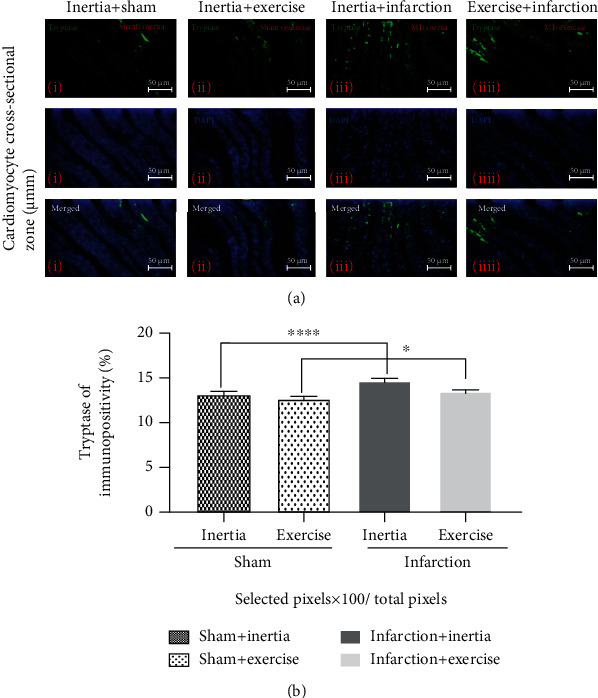
(a) Shows immunofluorescence micrographs of tryptase-positive mast cells in (i) sham+inertia, (ii) sham+exercise, (iii) infarction+inertia, and (iv) infarction+exercise groups. (b) Shows mean ± SEM of tryptase-positive mast cells in the studied group compared 3-way ANOVA, and Tukey tests; ^∗^*p* < 0.05 and ^∗∗∗∗^*p* < 0.0001.

Tryptase-positive MCs of sham+inertia and sham+exercise versus infarction+inertia and infarction+exercise groups expressed fewer tryptase (*p* = 0.0208). Also, MCs of sham+inertia and infarction+inertia versus sham+exercise and infarction+exercise groups expressed higher tryptase (*p* = 0.0001). As shown in Figure 5, many tryptase-positive MCs were observed in the infarction groups. In contrast, the expression of tryptase-positive MCs was less in the sham group (Figure 5).  Table 2 shows the distribution of low expression and high expression tryptase-positive MCs in the studied groups. Low expression and high expression tryptase-positive MCs of sham groups were significantly lower compared to infarction groups (*p* = 0.0208). Also, low expression and high expression tryptase-positive MCs of inertia groups were significantly higher than exercise groups (*p* = 0.0001).

## 4. Discussion

Briefly, in terms of the effect of exercise on cardiac fibrosis, we found lesser fibrosis in sham+exercise and infarction+exercise groups compared to sham+inertia and infarction+inertia groups, respectively (*p* = 0.023 and *p* = 0.001) (Figure 1). In the case of the effect of exercise on cardiomyocyte diameter, we observed cardiomyocyte diameter of infarction groups was significantly higher than sham groups (*p* < 0.05). At the same time, cardiomyocyte diameter of the infarction+exercise group was remarkably lesser than the infarction+inertia group (*p* < 0.05).

Regarding the effect of exercise on the total MC density and degranulation, while increased total MC density and degranulation occur at the site of fibrosis, we demonstrated aerobic exercise decreases both total MC density and degranulation in both sham and infarction groups (*p* < 0.05). Analysis of immunohistochemistry examination showed that there were significantly higher expressions of MCsʼ tryptase in infarction (inertia and exercise) groups than sham (inertia and exercise) groups (*p* < 0.05, *p* < 0.0001). Our regression analysis revealed the contradictory role of MCs in physiological-induced (sham) and pathological-induced (infarction) cardiac hypertrophy.

Despite advancements in the cure of MI in the previous years, occurrence and hospitalization rates are still growing [4]. Probes have collectively exposed that systematic practice is positive for the CV organ in adult, aged, normal, and unhealthy peoples. Therefore, practice has been suggested globally for CVD inhibition and cure. Though the advantages of practice are apparent, comprehension of the molecular causes that coordinate these impacts continues lacking and has been a subject of extreme investigation in current years [5]. MI is routinely connected with heart renovation (remodeling). Cardiac renovation is characterized as a collection of molecular, cellular, and interstitial alterations that are demonstrated in a clinical setting as alterations in extent, bulk, geometry, and role of the heart after damage. The course happens in a weak prognosis through its link with ventricular malfunction and diseased arrhythmias [6]. Here, inflammation and fibrosis are assumed to play fundamental actions. Throughout heart inflammation, immunity cells enter the heart and control tissue-damaging replies [7]. While proof shows that heart inflammation and fibrosis are probably reversible in animal models and medical settings, they are exciting goals for new heart failure management protocols. On this basis, animal models are vital as they simulate the medical settings of patients' cardiac MI [7]. The exact connection between inflammation and fibrosis continues to be ambiguous, but it seems that immune cells could encourage fibrosis by secreting fibrogenic elements. Nevertheless, beneficial methods aiming at inflammatory cells have yet been unsuccessful to change illness pathogenesis. A novel cell to meet search attention in fibrosis is the MC [8]. MCs discharge tryptase with proinflammatory properties, in reply to numerous signals [9]. MC maturing, phenotype, and action are a straight result of the native milieu and reply to numerous provocations over the secretion of a range of biologically dynamic intermediates. These characteristics enable MCs to play as both initial responders in damaging conditions as well as to reply to alterations in their milieu by connecting with various other cells. Thus, the censorious function of MCs, in both innate and adaptive immunity, has obtained amplified importance. On the other hand, MCs are contemplated as the key trespassers in numerous dysfunctions, autoimmune [10], and inflammatory diseases, including CV and metabolic illnesses and their related difficulties [11]. MCs exert their functional and pathological actions by discharging granules containing histamine, cytokines, chemokines, and proteases, including MCsʼ tryptases. Accordingly, since tryptase contributes to the pathogenesis of diabetes and its complexities, aiming MCs as a new remedy for diabetes needs more investigations [12]. In this context, it has been reported that the increased numbers of total MC density and degranulation in diabetic animals negatively affected the potentiometric properties of their skin wounds [13–15]. Recently, Wygrecka et al. have suggested that MCs might provide to the fibrotic course by encouraging local fibroblasts in the lung, therefore leading to the pathogenesis of the illness [16]. MCs have been frequently involved in the pathogenesis of heart fibrosis; however, these results are arguable [17]. Numerous animal probes have shown a shielding action for MCs. MC-deficient mice have been stated to have weaker results after coronary artery ligation and improved heart task upon MC reorganization [18]. On the contrary, MCs have also been incriminated as the main operators of fibrosis. MC stabilization throughout a hypertension animal simulation and an atrial fibrillation mouse simulation recovered related fibrosis [19]. Besides, cumulative data shows that stress deteriorates or advances coronary artery disease (CAD) by way of encouraging coronary MC, resulting in native inflammation. This impact might be more marked in sufferers with atherosclerosis or throughout acute MC degranulation by allergic or nonallergic activators. Merging anti-inflammatory and MC prohibiting modalities, as well as decrease of atherosclerosis and stress, might be a new cure approach [20].

Systematic practice partially decreases the danger of CVDs, since practice induces anti-inflammatory impacts. This impact might be partially mediated by stimulation of an anti-inflammatory milieu [21]. Accordingly, in terms of the impact of the practice on the total MC density and degranulation, while increased total MC density and degranulation occurs at the site of fibrosis, we demonstrated that aerobic exercise decreases both total MC density and degranulation in both sham and infarction groups (Figure 2).

On the other hand, Gleeson et al. reported that releasing of interleukin-6 (IL-6) from muscles under influence of exercise finally leads to a reduction of proinflammatory cytokine production, such as tryptase from MCs. In detail, Gleeson et al. explained that releasing of IL-6 from muscles into the circulation leads to an increase in circulating level of IL-10 and IL-1 receptor antagonist. A high level of IL-10-secreting regulatory T cells downregulates Toll-like receptor expression on monocytes and inhibits downstream responses and reduces proinflammatory cytokine creation, such as tryptase from MCs [21]. In the line with Gleeson et al.'s study, analysis of immunohistochemistry examination of the current study showed that there were significantly higher expressions of MCsʼ tryptase in infarction groups than sham groups. Moreover, there were significantly lesser expressions of MCsʼ tryptase in the infarction+exercise group than in the infarction+inertia group. Accordingly, low expression and high expression tryptase-positive MCs of sham groups were significantly lower compared to infarction groups (*p* = 0.0208). Also, low expression and high expression tryptase-positive MCs of inertia groups were significantly higher than exercise groups (*p* = 0.0001, Figure 5, Table 2). We found more tryptase in infarction groups, compared to sham groups. MC tryptase creates the active profibrotic structure of transforming growth factor-beta 1 (TGF-*β*1) from hidden structures secreted by MCs throughout activation, along with what is existing in the micro milieu [22]. TGF-*β*1 is key in fibrosis over encouragement of fibroblast activation, myofibroblast differentiation, and collagen production [7]. MCsʼ tryptase could directly prompt these activities on fibroblasts individualistically of TGF-*β*1 [23].

Wisløff et al. reported that aerobic durability exercise reduces the ventricular and myocyte hypertrophy and increases contractility in an animal model after MI [24]. Similar to Wisløff et al.'s findings, we observed that exercise increased cardiomyocyte size in the physiological range in both sham+exercise and infarction+exercise groups (Table 1). Moreover, in infarction groups, exercise significantly decreased the cardiomyocyte size of the exercise group compared to the inertia group.

We concluded that exercise improves cardiac function in both healthy rats and rats suffering from MI.

Lasting practice provokes renovation of the heart containing development and adaptive molecular and cellular reprogramming. Comparing the molecular and cellular basis of physiologic (sham groups of the current study) and pathologic (infarction groups of the current study) cardiac development has revealed phenotype-particular signaling paths and transcriptional controlling programs [25]. Probes recommend which practice paths presumably neutralize pathological paths, and practice is frequently suggested for sufferers with chronic stable heart failure or following MI [26]. Durability exercise creates the development of cardiomyocyte size with conserving contractile task. Importantly, exercise also neutralizes pathologic signaling in the situation of heart failure or offers heart protection from ischemic abuse [27].

## 5. Conclusion

Our conclusions are in rats who suffer from MI, aerobic exercise by inhibiting total MC density and degranulation, decreases MCsʼ tryptases and cardiac fibrosis, and improves cardiac function. Also, while there was increased total MC density, we could observe increased MCsʼ tryptases and cardiac fibrosis. Our regression analysis demonstrated the opposed actions of MCs in physiological-induced (sham) and pathological-induced (infarction) cardiac hypertrophy. Exercise improves cardiac function in both healthy rats and rats suffering from MI. A better understanding of MC roles and activities using MC stabilizers in rats suffering from MI under influence of aerobic exercise is needed to move forward in this field.

## Figures and Tables

**Table 1:** Echocardiographic and anatomical data of the 4 experimental groups at the end of the protocol.

Groups & number of rats⟶	Sham+inertia	Sham+exercise	Infarction+inertia	Infarction+exercise
Parameters↓				
*N*	10	9	8	10
Heart rate (bpm)	335 ± 7	332 ± 26	325 ± 17	332 ± 9
Fractional shortening (%)	51.0 ± 2.2	48.5 ± 1	23.7 ± 2.7^∗∗∗^	20.8 ± 2^∗∗∗^
Ejection fraction (%)	83.6 ± 1.7	72.0 ± 2.1	32.3 ± 2.1^∗∗∗^	39.5 ± 4.2^∗∗∗^
Interventricular septum in diastole (mm)	1.8 ± 0.1	1.7 ± 0.1	1.1 ± 0.1^∗∗∗^	1 ± 0.1^∗∗∗^
Left ventricular internal diameter in diastole (mm)	8.36 ± 0.1	8.3 ± 0.1	9 ± 0.3^∗^	10.9 ± 0.2^∗∗∗^
Systolic blood pressure (mmHg)	125 ± 4	120 ± 5	123 ± 2	125 ± 3
Diastolic blood pressure (mmHg)	80 ± 3	80 ± 1	81 ± 2	80 ± 1
Heart weight (g)	1.44 ± 0.01	1.75 ± 0.03^∗^	1.53 ± 0.04	1.60 ± 0.01^∗^
Body weight (g)	296 ± 12	326 ± 15^∗^	351 ± 13^∗^	352 ± 17^∗^
Heart weight/body weight	0.48 ± 0.01	0.55 ± 0.01^∗^	0.43 ± 0.01^∗^	0.45 ± 0.02^∗^
Cardiomyocyte diameter (*μ*m)	23 ± 2	38 ± 3^∗^	59 ± 2^∗^	43 ± 2^∗^

Values are means ± SE. Sham+inertia, sham+exercise, infarction+inertia, and infarction+exercise groups ^∗^*p* < 0.05, ^∗∗^*p* < 0.01, and ^∗∗∗^*p* < 0.001 vs. sham by 3-way ANOVA, and Tukey tests.

**Table 2:** Mean ± SEM of low expression and high expression tryptase-positive mast cells of studied groups compared by 3-way ANOVA and Tukey tests.

Groups	Mean ± SEM (low expression of tryptase)	Mean ± SEM (high expression of tryptase)
A. Sham+inertia	10.822 ± 0.41587	13.146 ± 0.33161
B. Sham+exercise	9.073 ± 0.34306	12.6 ± 0.33012
C. Infarction+inertia	13.146 ± 0.33161	14.52 ± 0.41
D. Infarction+exercise	12.6 ± 0.33012	13.38 ± 0.29

There were significant differences between A and B groups and C and D groups (*p* = 0.020). There were also significant differences between A and C groups and B and D groups (*p* = 0.001).

## Data Availability

The data used to support the study is available within the article and in the supplementary files (available here).

## Supplementary Materials

Supplementary Materials Table S1: effect of 7 weeks of exercise training after MI on percent of cardiac fibrosis. Table S2: effect of 7 weeks of exercise after MI on total density and activation of mast cells in cardiomyocytes or in the cardiomyocyte cross-sectional area. Table S3: relationship (correlations) between cardiomyocyte hypertrophy total cardiac MC and degranulated MC density. Table S4: relationships (correlations) between cardiac mast cell density (total) and degranulated mast cells on percent of cardiac fibrosis. Table S5: *p* value of findings of immunohistochemistry examination.

## References

[1] Melo S. F., Barauna V. G., Neves V. J. (2015). Exercise training restores the cardiac microRNA-1 and -214 levels regulating Ca2+ handling after myocardial infarction. *BMC Cardiovascular Disorders*.

[2] Nikooie R., Samaneh S. (2016). Exercise-induced lactate accumulation regulates intramuscular triglyceride metabolism via transforming growth factor-*β*1 mediated pathways. *Molecular and Cellular Endocrinology*.

[3] Kristensen C. M., Dethlefsen M. M., Tøndering A. S. (2018). PGC-1*α* in hepatic UPR during high-fat high-fructose diet and exercise training in mice. *Physiological Reports*.

[4] Zaprutko J., Michalak M., Nowicka A. (2017). Hospitalisation length and prognosis in heart failure patients. *Kardiologia Polska (Polish Heart Journal)*.

[5] Moreira J. B. N., Wohlwend M., Wisløff U. (2020). Exercise and cardiac health: physiological and molecular insights. *Nature Metabolism*.

[6] Azevedo P. S., Polegato B. F., Minicucci M. F., Paiva S. A., Zornoff L. A. (2016). Cardiac remodeling: concepts, clinical impact, pathophysiological mechanisms and pharmacologic treatment. *Arquivos Brasileiros de Cardiologia*.

[7] Prabhu S. D., Frangogiannis N. G. (2016). The biological basis for cardiac repair after myocardial infarction: from inflammation to fibrosis. *Circulation Research*.

[8] Overed-Sayer C., Rapley L., Mustelin T., Clarke D. L. (2014). Are mast cells instrumental for fibrotic diseases?. *Frontiers in Pharmacology*.

[9] Gri G., Frossi B., D’Inca F. (2012). Mast cell: an emerging partner in immune interaction. *Frontiers in Immunology*.

[10] da Silva E. Z. M., Jamur M. C., Oliver C. (2014). Mast cell function: a new vision of an old cell. *Journal of Histochemistry & Cytochemistry*.

[11] Xu J.-M., Shi G.-P. (2012). Emerging role of mast cells and macrophages in cardiovascular and metabolic diseases. *Endocrine Reviews*.

[12] Hamdy N., Salam R. F., Mohamed N. A. E.-G. (2018). Mast cell, a new player in type 2 diabetes. *Kasr Al Ainy Medical Journal*.

[13] Bagheri M., Amini A., Abdollahifar M. A. (2018). Effects of photobiomodulation on degranulation and number of mast cells and wound strength in skin wound healing of streptozotocin-induced diabetic rats. *Photomedicine and Laser Surgery*.

[14] Kouhkheil R., Fridoni M., Abdollhifar M. A. (2019). Impact of photobiomodulation and condition medium on mast cell counts, degranulation, and wound strength in infected skin wound healing of diabetic rats. *Photobiomodulation, Photomedicine, and Laser Surgery*.

[15] Soleimani H., Amini A., Abdollahifar M.-A. (2021). Combined effects of photobiomodulation and curcumin on mast cells and wound strength in wound healing of streptozotocin-induced diabetes in rats. *Lasers in Medical Science*.

[16] Wygrecka M., Kwapiszewska G., Jablonska E. (2011). Role of protease-activated receptor-2 in idiopathic pulmonary fibrosis. *American Journal of Respiratory and Critical Care Medicine*.

[17] Legere S. A., Haidl I. D., Légaré J. F., Marshall J. S. (2019). Mast cells in cardiac fibrosis: new insights suggest opportunities for intervention. *Frontiers in Immunology*.

[18] Shao Z., Nazari M., Guo L. (2015). The cardiac repair benefits of inflammation do not persist: evidence from mast cell implantation. *Journal of Cellular and Molecular Medicine*.

[19] Levick S. P., McLarty J. L., Murray D. B., Freeman R. M., Carver W. E., Brower G. L. (2009). Cardiac mast cells mediate left ventricular fibrosis in the hypertensive rat heart. *Hypertension*.

[20] Alevizos M., Karagkouni A., Panagiotidou S., Vasiadi M., Theoharides T. C. (2014). Stress triggers coronary mast cells leading to cardiac events. *Annals of Allergy, Asthma & Immunology*.

[21] Gleeson M., Bishop N. C., Stensel D. J., Lindley M. R., Mastana S. S., Nimmo M. A. (2011). The anti-inflammatory effects of exercise: mechanisms and implications for the prevention and treatment of disease. *Nature Reviews Immunology*.

[22] Tatler A. L., Porte J., Knox A., Jenkins G., Pang L. (2008). Tryptase activates TGF*β* in human airway smooth muscle cells via direct proteolysis. *Biochemical and Biophysical Research Communications*.

[23] Abe M., Kurosawa M., Ishikawa O., Miyachi Y., Kido H. (1998). Mast cell tryptase stimulates both human dermal fibroblast proliferation and type I collagen production. *Clinical and Experimental Allergy: journal of the British Society for Allergy and Clinical Immunology*.

[24] Wisløff U., Loennechen J. P., Currie S., Smith G. L., Ellingsen Ø. (2002). Aerobic exercise reduces cardiomyocyte hypertrophy and increases contractility, Ca2+ sensitivity and SERCA-2 in rat after myocardial infarction. *Cardiovascular Research*.

[25] Emter C. A., McCune S. A., Sparagna G. C., Radin M. J., Moore R. L. (2005). Low-intensity exercise training delays onset of decompensated heart failure in spontaneously hypertensive heart failure rats. *American Journal of Physiology-Heart and Circulatory Physiology*.

[26] Pandey A., Parashar A., Kumbhani D. J. (2015). Exercise training in patients with heart failure and preserved ejection fraction: meta-analysis of randomized control trials. *Circulation: Heart Failure*.

[27] Vega R. B., Konhilas J. P., Kelly D. P., Leinwand L. A. (2017). Molecular mechanisms underlying cardiac adaptation to exercise. *Cell Metabolism*.

