# Supplementary material for: Aerobic Exercise-Assisted Cardiac Regeneration by Inhibiting Tryptase Release in Mast Cells after Myocardial Infarction
Source: Biomed Res Int. 2021 Jun 8;2021:5521564. doi: 10.1155/2021/5521564 (PMC8205576; doi:10.1155/2021/5521564)
Supplement: Supplementary Materials — Table S1: effect of 7 weeks of exercise training after MI on percent of cardiac fibrosis. Table S2: effect of 7 weeks of exercise after MI on total density and activation of mast cells in cardiomyocytes or in the cardiomyocyte cross-sectional area. Table S3: relationship (correlations) between cardiomyocyte hypertrophy total cardiac MC and degranulated MC density. Table S4: relationships (correlations) between cardiac mast cell density (total) and degranulated mast cells on percent of cardiac fibrosis. Table S5: p value of findings of immunohistochemistry examination. [file 5521564.f1.docx]

**Data in supplementary information files for** **manuscript ID No. 5521564**

**Aerobic exercise-assisted cardiac regeneration by inhibiting tryptase release in mast cells after myocardial infarction.**

1. The Exercise protocol and echocardiography data used to support the findings of this study are included within the article

2. The animal data used to support the findings of this study are included within the article.

3. The Immunohistochemistry analysis Exercise protocol and echocardiography data used to support the findings of this study are included within the supplementary information file(s).

4. The [code of research] data used to support the findings of this study were supplied by [Ethical] under license and so cannot be made freely available. Requests for access to these data should be made to [LUMS.REC.1396.257].

5. The [Approve the plan] data used to support the findings of this study may be released upon application to the [Vice Chancellor of Research and Technology at Lorestan University of Medical Sciences, Khorramabad, Iran], who can be contacted at [00986633120133].

6. Previously reported [Article] data were used to support this study and are available at [DOI: 10.4149/BLL_2020_003].

7. The data used to support the findings of this study are available from the corresponding author upon request. Below is a sample data that viewers may use for their research work.

**Table S1. Effect of 7 weeks of exercise training after MI on percent of Cardiac fibrosis.**

| GROUPS | Cardiac fibrosis (%) | P(value) |
| --- | --- | --- |
| Sham+inertia | 6.2±0.6 | Sham+inertia versus (p=0.023) |
| Sham+ exercise | 2.2±0.8 |  |
| Infarction+inertia | 14.5±1.1% | Sham+ exercise versus  Infarction+exercise(p=0.001) |
| Infarction+exercise | 8.8±1.2 % |  |

**Table S2. Effect of 7 weeks of exercise after MI on total density and activation of mast cells in cardiomyocytes or** **in the cardiomyocytes cross-sectional area.**

| GROUPS | Total Mast Cells(cell/mm^2^)  Data & (P values) | Degranulated mast cells(cell/mm2)  Data& (P values) |
| --- | --- | --- |
| Sham+inertia | 1.5±0.23, p=0.0063 | 48±2, p=0.0013 |
| Sham+ exercise | 1.2±0.31, p=0.0099 | 58±3.0, p=0.0042 |
| Infarction+inertia | 3.4±0.22, p=0.0099 | 67±3, p=0.0013 |
| Infarction+exercise | 3.1±0.20, p=0.0244 | 52±1.5, p=0.0117 |

**Table S3. Relationship (correlations) between** **cardiomyocytes hypertrophy total cardiac MC and degranulated MC density.**

| GROUPS | Total Mast Cells(cell/mm^2^)  regression(r^2^), P values | Degranulated mast cells(cell/mm2)  regression(r^2^), P values |
| --- | --- | --- |
| cardiomyocytes hypertrophy in Sham | r^2^=0.555.8  p=0.0001 | r^2^=0.6150  p=0.0001 |
| cardiomyocytes hypertrophy in  Infarction | r^2^=0.1634  p=0.0423 | r^2^=0.1367  p=0.0312 |

**Table S4.** **Relationships (correlations) between cardiac mast cells density (total) and degranulated mast cells on percent of cardiac fibrosis.**

| GROUPS | Total Mast Cells(cell/mm^2^)  regression(r^2^), P values | Degranulated mast cells(cell/mm2)  regression(r^2^), P values |
| --- | --- | --- |
| percent of cardiac fibrosis in Sham | r^2^=0.1706  p=0.0155 | r^2^=0.1847  p=0.0108 |
| percent of cardiac fibrosis in  Infarction | r^2^=0.117  p=0.1012 | r^2^=0.2083  p=0.03386 |

**Table S5. pvalue of Findings of immunohistochemistry examination**

| GROUPS | Mean ±SE (low expression of tryptase) | Mean ±SE ( high expression of tryptase) |
| --- | --- | --- |
| **A**, Sham+inertia | 10/822± 0/41587 | 13/146 ±0/33161 |
| **B**, Sham+ exercise | 9/073± 0/34306 | 12/6± 0/33012 |
| **C**, Infarction+inertia | 13/146± 0/33161 | 14/52 ±0/41 |
| **D, I**nfarction+exercise | 12/6± 0/33012 | 13/38 ±0/29 |

**(AB vs CD) p=0/0208 * Yes**

**(AC vs BD) p=0/0001 **** Yes**
